# Diastasis of the Sternum

**Published:** 1877-10-20

**Authors:** 


Diastasis of the Sternum by the Violent Action of the Diaphragm During Coughing. By F. J. Lutz, M. D. An interesting paper touching an affection not generally known or understood.

